# Identification of enterobacteriaceae causing septicemia in the axolotl *Ambystoma mexicanum*

**DOI:** 10.1007/s10482-025-02067-y

**Published:** 2025-03-01

**Authors:** Osvaldo Lopez Diaz, Antonio Buendia, Joaquín Sánchez, Guiehdani Villalobos, Nora Rojas-Serrania, José Antonio Ocampo Cervantes, Fernando Uranga-Muñoz, Fernando Martinez-Hernandez, Emilio Rendón-Franco, Claudia Irais Muñoz-García

**Affiliations:** 1 Departamento de Producción Agrícola y Animal, Universidad Autónoma Metropolitana, Unidad Xochimilco, 04960 Mexico City, México; 2 Veterinary Faculty, Universidad de Murcia, Murcia, Spain; 3 Centro de Investigaciones Biológicas y Acuícolas de Cuemanco (CIBAC), Universidad Autónoma Metropolitana - Unidad Xochimilco, Mexico City, México; 4 Hospital General “Dr. Manuel Gea González”, Mexico City, México

**Keywords:** Amphibian, Aquatic, Enterobacteria, Salamander, Sepsis

## Abstract

The *Ambystoma mexicanum* axolotl is a highly threatened amphibian and a valuable research model, with very little information about bacterial diseases affecting it. The aim of this study was to perform an identification of bacteria responsible for septicemia in three individuals. For all of them, necropsies were made, bacteria classification was performed by traditional and DNA-based molecular methods and tissues were histologically examined. All animals showed edema and ascites, and other tissues such as the lungs, spleen, liver, and kidney were also affected, dermatitis also occurred, in one case, the dermatitis was severe. Two bacterial isolates showed genetic identities of 99% with *Aeromonas veronii*, one with *Citrobacter freundii*, and another with 100% identity with *Citrobacter portucalensis*. These and other Enterobacteriaceae species of *Aeromonas* genus have been reported to produce septicemia in Anura amphibians and fish, pointing out that they are a health hazard for aquatic animals. Future endeavors to determine these bacteria prevalence, the search for antibiotic resistance/susceptibility, factors that can trigger the pathology, and the development of early diagnostic tools should be done to improve our understanding.

## Introduction

The axolotl (*Ambystoma mexicanum*) is a microendemic neotenic salamander, and a valuable research model widely used due to its regenerative capabilities (Gresens 2004). Unfortunately, wild populations face threats because of habitat loss and degradation, making it a highly threatened amphibian, listed as critically endangered by the IUCN (2020) and at risk of extinction by Mexican laws (Semarnat 2010). However, other adverse causes such as infectious diseases may have been overlooked, even when it is known that this is the second more important threat for Mexican amphibians (Frías-Alvarez et al. 2010).

Nowadays, some strategies as managed-breeding and reintroduction programs are on-going to save this species from extinction (Smith and Rojas 2023). And given that reintroduction is a complex process which must include diseases surveillance and potential pathogens identification, thus information is necessary to avoid failure of post-release survival or risk of introducing diseases and pathogens into nature (Linhoff et al. 2021). Unfortunately, for this species studies about illness and mortality and its etiological determination have been barely realised.

Among infectious pathogens, bacteria are common in ex situ amphibian populations; and one of the most reported was the red-leg syndrome, which is a multifactorial disorder with an important role of bacteria (Densmore and Green 2007). Specifically, for *A. mexicanum* bacterial dermatitis is reported as the most common disease (Takami and Une 2017) but, no comprehensive studies have been conducted to identify specific etiological agents, except for a register of *Bacillus cereus* causing septicemia (Huang et al. 2022). Interestingly, in captive amphibian bacterial infections have been attributed to crowded living conditions and poor water quality which leads to physiological stress. In the Xochimilco water channels, the remnant natural area for its reintroduction, conditions such as contamination and coexistence with exotic species under crowding conditions could favor similar conditions as in captivity, leading also to physiological stress. Thus, information on etiology-specific mortality is needed for any ex-situ and in-situ conservation program to improve the axolotl survival.

The aim of this study was to perform the identification of bacteria responsible for septicemia in axolotls, maintained under similar ex situ semi-natural water conditions.

## Materials and methods

### Studied animals

Animals used in this study were part of the 2022 captive population of 300 individuals of the research center “Centro de Investigaciones Biológicas y Acuícolas de Cuemanco (CIBAC)”. This center is located within the Xochimilco channels in Mexico City, 19°16′54.875″ N 99°6′7.788″ W. The CIBAC´s population individuals descended from Xochimilco axolotl’s founders captured in the area during 2007 (Smith and Rojas 2023). This study was performed on three animals; all of them were raised and maintained under the same conditions. The animals in this study were adults, one of them was a female of the melanic/wild-type variety and the other two were males of leucistic-type (henceforth referred to as LT1 and LT2). Axolotls were kept indoor in individual plastic containers filled with ten liters of water from the adjacent wetland—the Xochimilco channels, filtered by a constructed wetland, aerated continuously with an electric-powered air pump and with weekly water changes. Animals were fed ad libitum with *Tubifex* sp. and were daily checked.

Between March 31 and July 26 of 2022 the three aforementioned animals were found freshly dead, without post-mortem changes at first gross inspection, and with no previous history of illness. For each one necropsies were performed, swab samples were taken from ascites fluid, lungs and/or gills under sterile conditions for bacterial detection using brain heart infusion broth (BHI). Finally, complete incised specimens were fixed in 10% neutral-buffered formalin for histological examination.

### Histopathological analysis

Paraffin-embedded tissue blocks were prepared, and 4-μm thick tissue sections were cut and stained with hematoxylin and eosin (HE) and Sandiford stain.

### Microbiological analysis

Collected swab samples were initially incubated at 37 °C for 24 h into the enrichment media BHI, then 25 μL of bacterial suspension was seeded onto blood agar and McConkey agar at 37 °C for 24 h. Initially, the identification of bacterial species was based in Gram stain reaction and, hemolysis detection and classification on blood agar. Then, biochemical tests performed were oxidase, triple-sugar iron, sulfide-indole motility, sodium citrate and urea incubated at 37 °C for 24 h, except sodium citrate and urea that were incubated for up to 7 days.

Each cultured and identified bacterium was collected from the plate and put into 1.5 mL of 10% skim milk (Svelty, Nestlé®) and glycerin, and stored at − 20 ºC.

### Molecular analysis

The DNA isolation was established with 100 µL of each bacterium-milk suspension and homogenised with 400 µL of lysis solution (50 mM Tris–HCl, 50 mM EDTA, pH 8, 50 mM NaCl, 1% SDS and 20 μg/mL Proteinase K) and incubated at 55 °C overnight. The phenol–chloroform technique was used to extract DNA (Sambrook et al. 2001).

PCR was performed on a Veriti 96 well thermal cycler (Applied Biosystems®), using 1X PCR buffer (8 mM Tris–HCl, pH 8, 20 mM KCl), 2 mM MgCl_2_, 2 µL of BSA (2 mg/mL), 0.5 mM dNTPs, 2U of *Taq DNA Polymerase* (Invitrogen®, 11,615–036), 1 mM of universal primers for the 16S rRNA gene (27F: 5´-AGA GTT TGA TCM TGG CTCA G-3´ and 1492R: 5´-GGT TAC CTT GTT ACG ACT T-3´), 500 ng of genomic DNA, and adjusted with sterile distilled water to a volume of 50 µL of reaction. Amplification conditions were one cycle at 94 °C for 5 min, 35 cycles including denaturation, annealing and extension steps at 94 °C −45 s, 54 °C-1.30 min and 72 °C-1.30 min, respectively with final extension at 72 °C for 7 min. The presence of amplicons was checked by electrophoresis in 1.5% agarose gel, after which the band was purified using AxyPrep PCR clean-up kit (Axigen Biosciences®, CA, USA) and sequenced on both strands by a commercial supplier. The sequences were deposited in GenBank under accession numbers: ON203096, MT345040, OQ692577 and AP022378.

Multiple alignments were performed with the CLUSTAL W program v1.8 (Thompson et al. 1994) in MEGA program v4 (Kumar et al. 2004), while the ModelTest 3.7 program (Posada and Crandall 1998) was used to determine the appropriate model of molecular evolution. The sequences were analysed with the general time-reversible model with gamma distribution. A phylogenetic reconstruction using Bayesian inference was performed with the program Mr. Bayes 3.1.2 (Ronquist and Huelsenbeck 2003). The analysis was executed for 2 million generations. Sampling trees were built every 100 generations, and those with scores lower than the stationary phase were discarded, while those that reached the stationary phase were collected and used to build a consensus tree.

## Results

**Fig. 1:**
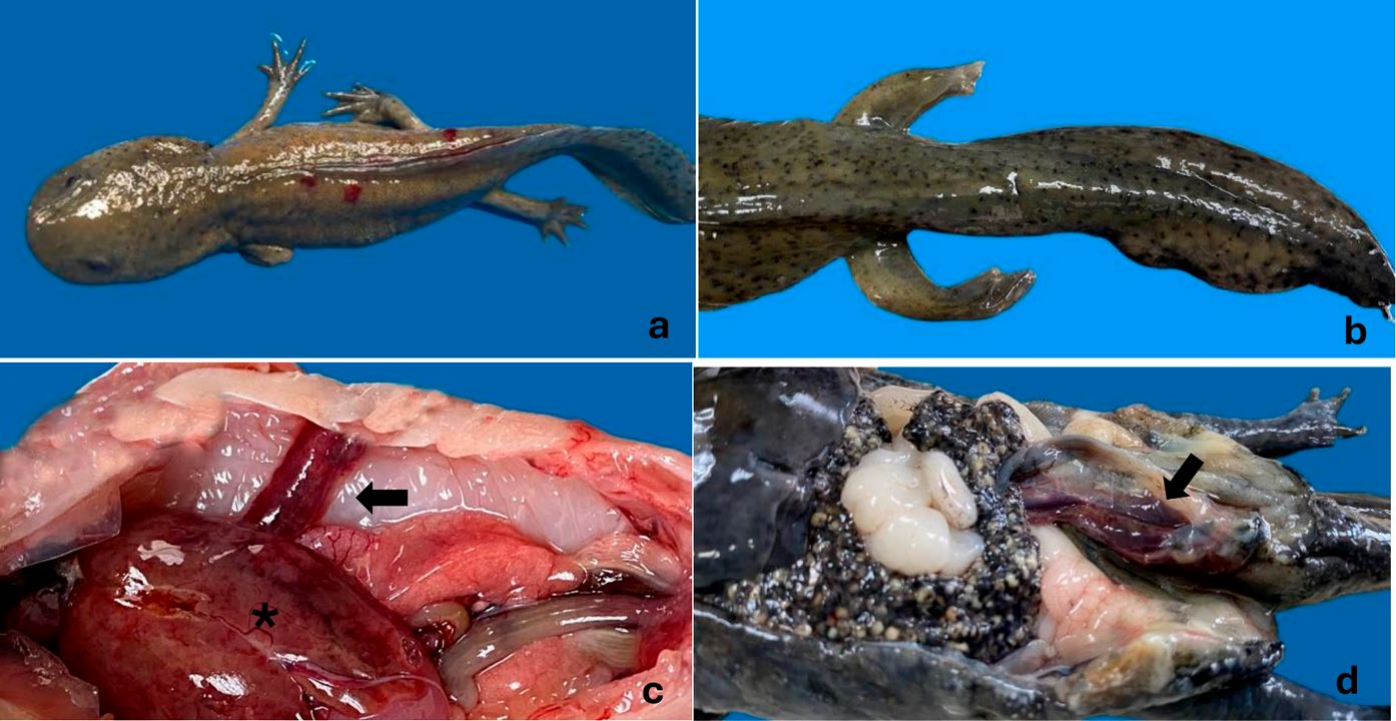
**a** Dermatitis ulcerative, **b** Pelvic limb edema, **c** Lung redness (arrow) and hepatomegaly (asterisk), **d** Kidney enlargment (arrow)

For the studied individuals macroscopical gross lesions were observed in the skin, pelvic limbs, cloaca, lungs, spleen, and liver. Lesions at these locations were found on at least one of the studied specimens. For LT2 and melanic individuals skin ulcers exist (Fig. 1a) and discreet skin hemorrhages were observed on LT1. All animals showed pelvic limbs with moderate edema (Fig. 1b) and, the LT2 showed cloacal swelling. Ascites was seen in all axolotls, ranging from mild to moderated. The lungs showed varying degrees of hyperemia (Fig. 1c), from mild to severe, while LT2 also showed diffuse unilateral hyperinflation. Hepatomegaly was present in LT1 individual (Fig. 1c). The liver was pale brown and firm to the touch, with discrete multifocal reddish areas in the LT2. And for melanic and LT1 individuals the liver was mildly, diffusely brown discolored, with streaks and multifocal to coalescing reddish foci and slightly friable to palpation. In the three axolotls, moderate blood leakage was consistently observed when the liver was incised. Other organs affected were the spleen and kidney, splenomegaly with marked hyperemia was observed in LT1, while the kidney of melanic individual showed enlargement and congestion (Fig. 1d).

**Fig. 2:**
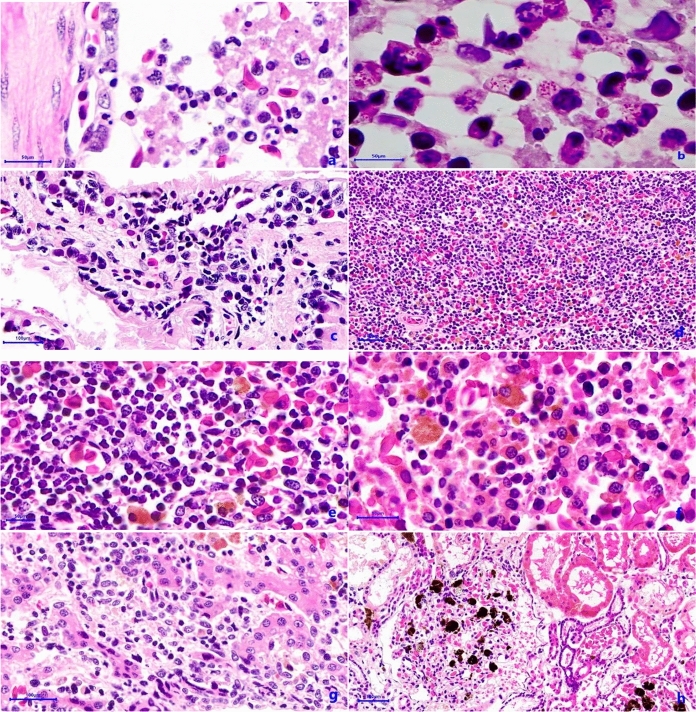
**a** Purulent pneumonia HE, 400x, **b** Macrophages with intracytoplasmic Gram-negative bacilli. Sandiford stain 1000x, **c** Granulomatous pneumonia HE, 400x, **d** Lymphoid depletion HE, 100x, **e** Purulent splenitis HE, 400x, **f** Necrotic hepatitis with mixed infiltrate HE, 400x, **g** Interstitial hepatitis HE, 400x, **h** Glomerulonephritis HE, 100x

The histopathological findings were, LT2 presented purulent pneumonia with intracytoplasmic presence of Gram-negative bacilli inside neutrophils and macrophages (Fig. 2a and 2b), and for the two other, granulomatous pneumonia was seen (Fig. 2c). In the liver, melanic individual presented necrotic hepatitis (Fig. 2f) and the other two had granulomatous hepatitis. All axolotls showed different degrees of lymphocytic or necrotic gastritis and enteritis, plus lymphoid depletion (Fig. 2d), and LT2 had purulent splenitis (Fig. 2e). At the renal level, the three of them presented glomerulonephritis (Fig. 2h) and renal congestion.

Based on microbiological, gross, and histopathological findings, the cause of death of the three axolotls was established as septicemia.

**Fig. 3:**
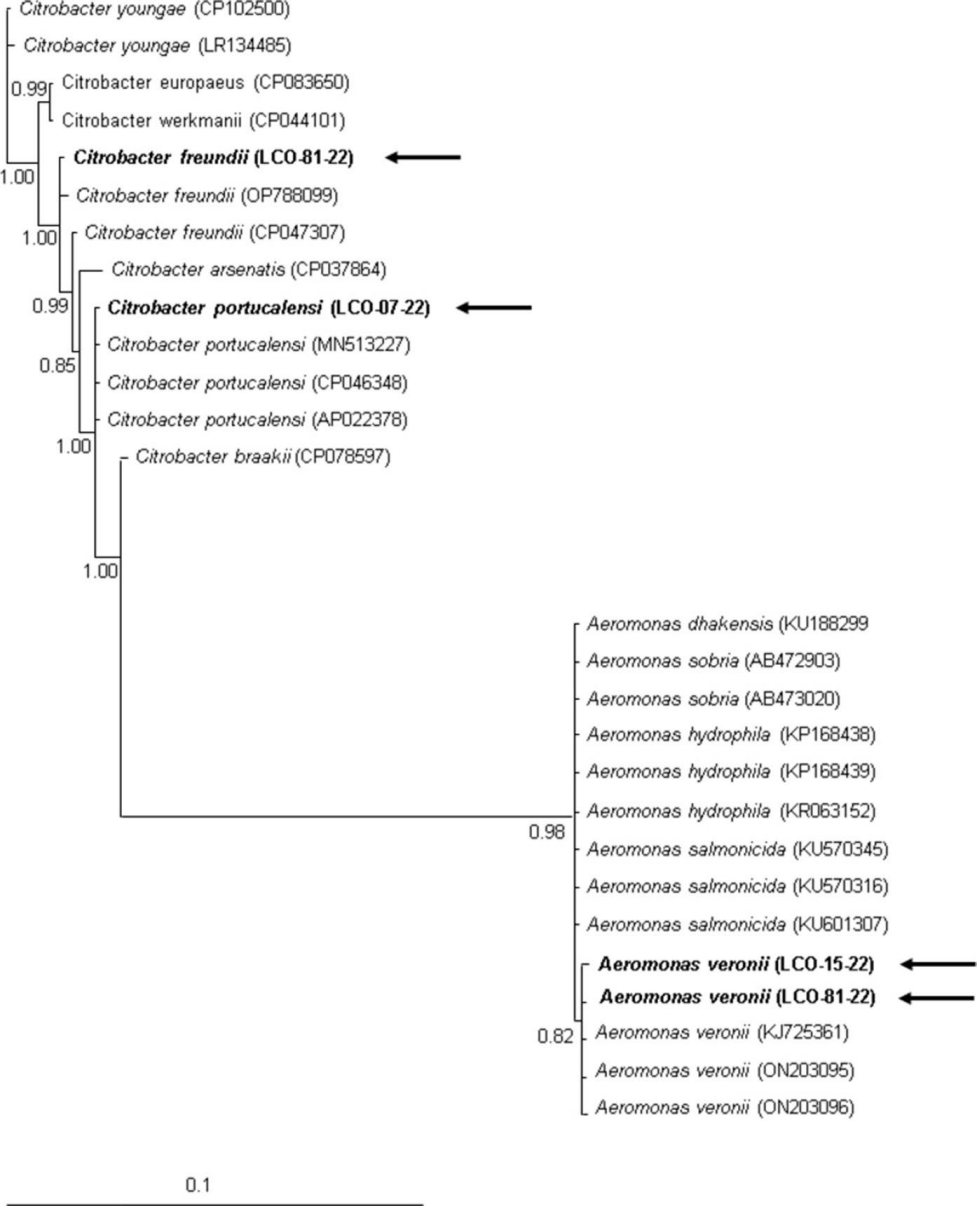
Bayesian tree using 16S partial sequence of *Aeromonas,* and *Citrobacter* sp. The numbers in nodes mean the posterior probability values. GenBank accession numbers are shown at the end of each branch, The sequences obtained in this study were identified by an arrow and bold letters

Seven bacterial isolates were obtained from ascitic fluid, lungs and/or gills, and were identified as *Salmonella arizonae* and *Aeromonas* spp. Four isolates were selected for molecular identification through PCR-sequencing using the 16S ribosomal DNA unit (Fig. 3).

According to the BLAST analyses in NCBI, two of the sequences of the isolates showed genetic identities of 99% (1126/1127) with *Aeromonas veronii* (ON203096 or MT345040), another sequence correlated with *Citrobacter freundii* (OQ692577) with identities of 99% (1125/1126) and the last sequence with an identity of 100% (1126/1126) with *Citrobacter portucalensis* (AP022378). The phylogenetic analyses showed identical results grouping the previous sequences into the respective clades of *A. veronii, C. freundii,* and *C. portucalensis* with high support values (above 0.82). Bacterial species identified per individual and fluid/anatomical site of isolation are shown in Table 1.

**Table 1:** Bacterial species identified in the three septicemic axolotls

Individual	Tissue/Body fluid source	Bacterial species identified by	
		Morphology and biochemical tests	Molecular methods
Melanic	Ascites fluid	*Salmonella arizonae*	*Citrobacter portucalensis* (AP022378)
	Lungs	*Aeromonas* sp.	*Aeromonas veronii* (ON203096)
LT1	Ascites fluid	*Salmonella arizonae*	*Citrobacter freundii* (OQ692577)
		*Aeromonas* sp.	*
LT2	Ascites fluid	*Aeromonas* sp.	*Aeromonas veronii* (MT345040)
		*Salmonella arizonae*	*
	Gills	*Salmonella arizonae*	*

*No molecular data

*LT1*: leucistic type one, *LT2*: leucistic type two.

## Discussion

The septicemia is a common lethal syndrome to amphibians, especially for those under human care (Densmore and Green 2007). However, septicemia is mainly reported in Anura and barely in Caudata species (Parto et al. 2014). Although, this disease caused by *Citrobacter* spp. and *Aeromonas* spp. is common and relevant for amphibians, bacterial dermatitis is the most prevalent disease in axolotls, to date no etiological study has been conducted thus, this is the first report.

Regarding the pathological effects of amphibian septicemia, authors report multi-organ damage characterised by congestion, inflammation and enlargement of the liver, spleen, and kidney, plus skin erythema and erosion, skin and subcutaneous edema and ascites (Pasteris et al. 2006; Parto et al. 2014); what shows that our pathological results in the axolotls were very similar to those previously reported in other amphibian species. Interestingly, according to previous reports it seems that the most common antemortem sign for amphibians is dermatitis, which is evident by skin redness; but unfortunately, in this study neither the leucistic or melanic axolotls was possible to detect, probably because the skin redness is not severe in leucistic axolotls and is difficult to see in dark skin individuals (melanic/wild type), therefore other methods should be explored for early diagnostic.

The bacteria isolated here, *Aeromonas veronii*, *Citrobacter freundii* and *C. portucalensis*, from axolotls were commonly reported in fish aquaculture causing pathology (Tekedar et al. 2019; Liu et al. 2024) and perhaps it is also common in Anura amphibians since some cases have already been reported (Pasteris et al. 2011; Edery et al. 2021; Zepeda-Velazquez et al. 2023). In Mexico, these bacteria have been identified in American bullfrogs (*Rana (Aquarana*) *catesbeiana*) in captivity with some evidence of disease, being *A. veronii* the second more prevalent, *C. freundii* the fifth and *C. portucalensis* isolated just once (Zepeda-Velazquez et al. 2023). However, it should be considered that bacteria not always were isolated from pathological tissues (Zepeda-Velazquez et al. 2023). For this axolotl population, a study of characterisation of skin microbiota in non-pathological individuals shows a single sequence of Enterobacteriaceae family, corresponding to a species of *Escherichia-Shigella* (Soto-Cortés et al. 2024); suggesting that these three are exogenous bacteria species, however a deeper study will be carried out.

Of the three Enterobacteriaceae bacteria detected here, perhaps the most reported was *Aeromonas*, which has a wide geographical distribution and is common in heterotherm animals, such as fish (Janda and Abbott 2010). In fact, septicemia has been attributed mainly to *Aeromonas hydrophila* (Pessier 2017), however, current diagnostic tools shows that other *Aeromonas* species have been implicated, such is the case here of *A. veronii*. The *Aeromonas* genus is commonly found in aquatic environments and have a great impact on aquaculture (Tekedar et al. 2019). Even, most of these bacteria pathological information has been generated in fish, including septicemia and skin lesions (Wang et al. 2022); in fact, the reported lesions were similar to those observed here in axolotls. Particularly in amphibians, the records of natural acute septicemia have been done in American bullfrogs under human care and were corroborated using experimental infections. The gross lesions in American bullfrogs included liver, spleen and kidney enlargement, intestinal edema and inflammation, and ascitic fluid; and histopathology showed hepatic and renal necrosis and degeneration, white pulp degeneration of the spleen and, congestion and villus shedding (Lin et al. 2024). Again, all these findings are compatible with those found here in the axolotls.

The *Citrobacter* genus is close related to *Salmonella* and *Escherichia coli*. These bacteria are known for being also important pathogens in aquaculture, being *C. freundii* the most representative species (Cortés-Sánchez et al. 2023; Sai et al. 2023). Interestingly, *Citrobacter freundii* is capable to infect and produce pathogenic effects in the American bullfrog, being the liver and spleen the most affected tissues (Pasteris et al. 2006). Histology showed necrotic lesion in liver and spleen with leucocyte infiltration, and for liver, decrease in melanin-containing cells was reported (Yang et al. 2024). Necrotic focus and leucocyte infiltration was not observed in the axolotls of this study, but these lesions were found in other axolotl organs, like intestine and lungs. And besides, perhaps the liver discoloration found in the present could be related to decrease in melanin-containing cells, as it happened in the American bullfrog (Yang et al. 2024).

*Citrobacter portucalensis* is a recently described *Citrobacter* species (Ribeiro et al. 2017), however, the interest in this bacterium is growing sharply (Sellera et al. 2022). Its isolation from sick amphibians occurs recently, with a low prevalence compared to *C. freundii*, and its pathology is not described yet (Zepeda-Velazquez et al. 2023). However, the finding of this bacterium in septicemic axolotl point to it as a potential threat.

Given that bacteria species frequently isolated from aquatic environments can lead to septicemia in axolotls, future endeavors to understand them should be made; including to determine its prevalence at population level, looking for antibiotic resistance/susceptibility and in this way narrow-spectrum treatments, and the develop of early diagnostic tools. Since mass mortality have been not reported in the axolotls, it is also important to evaluate all factors that can trigger the septicemia pathology, others than the mere presence of these bacteria species.

## Data Availability

No datasets were generated or analysed during the current study.

## References

[CR1] Cortés-Sánchez ADJ, Salgado-Cruz MDLP, Diaz-Ramírez M, Torres-Ochoa E, Espinosa-Chaurand LD (2023) A review on food safety: the case of *Citrobacter* sp. Fish and Fish Products Appl Sci 13:6907. 10.3390/app13126907

[CR2] Densmore CL, Green DE (2007) Diseases of amphibians. ILAR J 48:235–254. 10.1093/ilar.48.3.235 17592186 10.1093/ilar.48.3.235

[CR3] Edery S, Elias R, Shiva C, Weaver T, Reading R (2021) Cutaneous bacteria of confiscated *Telmatobius culeus* in Lima, Peru. J Wildl Dis 57:900–902. 10.7589/JWD-D-20-00076 34424988 10.7589/JWD-D-20-00076

[CR4] Frías-Alvarez P, Zúñiga-Vega JJ, Flores-Villela O (2010) A general assessment of the conservation status and decline trends of Mexican amphibians. Biodivers Conserv 19:3699–3742. 10.1007/s10531-010-9923-9

[CR5] Gresens J (2004) An introduction to the Mexican axolotl (*Ambystoma mexicanum*). Lab Anim 33:41–47. 10.1038/laban1004-41 10.1038/laban1004-41 15457201

[CR6] Huang XH, Li SY, Zhan GP, Li MR, Li YX, Huang ZJ, Wang L, Yin GW (2022) Isolation, identification and pathogenicity analysis of *Bacillus cereus* from *Ambystoma mexicanum*. Chi Vet Sci 52:1149–1156

[CR7] IUCN SSC Amphibian Specialist Group. 2020. *Ambystoma mexicanum*. The IUCN Red List of Threatened Species 2020: e.T1095A53947343. 10.2305/IUCN.UK.2020-3.RLTS.T1095A53947343.en. Accessed 30 October 2024

[CR8] Janda JM, Abbott SL (2010) The genus Aeromonas: taxonomy, pathogenicity, and infection. Clin Microbiol Rev 23:35–73 20065325 10.1128/CMR.00039-09 PMC2806660

[CR9] Kumar S, Tamura K, Nei M (2004) MEGA3: integrated software for molecular evolutionary genetics analysis and sequence alignment. Brief Bioinform 5:150–163. 10.1093/bib/5.2.150 15260895 10.1093/bib/5.2.150

[CR10] Lin H, Zeng G, Yu Y, Li H, Chen K, Qin Z, Jiang B, Li W, Su Y, Lin L, Liu C (2024) Acute septicemia and diagnostic evaluation of *Aeromonas veronii* infection in American bullfrogs (*Aquarana catesbeiana*). Aquaculture 580:740349. 10.1016/j.aquaculture.2023.740349

[CR11] Linhoff LJ, Soorae PS, Harding G, Donnelly MA, Germano JM, Hunter DA, McFadden M, Mendelson III JR, Pessier AP, Sredl MJ, Eckstut ME (eds) IUCN Guidelines for amphibian reintroductions and other conservation translocations. Gland, Switzerland: IUCN. 2021. https://portals.iucn.org/library/sites/library/files/documents/2021-017-En.pdf. Accessed 30 October 2024

[CR12] Liu J, Pan Y, Jin S, Zheng Y, Xu J, Fan H, Khalid M, Wang Y, Hu M (2024) Effects of *Citrobacter freundii* on sturgeon: insights from skin mucosal immunology and microbiota. Fish Shellfish Immunol 149:109527. 10.1016/j.fsi.2024.109527 38561068 10.1016/j.fsi.2024.109527

[CR13] Parto P, Haghighi ZM, Vaissi S, Sharifi M (2014) Microbiological and histological examinations of endangered *Neurergus kaiseri* tissues displaying red-leg syndrome. Asian Herpetol Res 5:204–208

[CR14] Pasteris SE, Bühler MI, Nader-Macías ME (2006) Microbiological and histological studies of farmed-bullfrog (*Rana catesbeiana*) tissues displaying red-leg syndrome. Aquaculture 251:11–18. 10.1016/j.aquaculture.2005.05.007

[CR15] Pasteris SE, Guidoli MG, Otero MC, Bühler MI, Nader-Macías ME (2011) *In vitro* inhibition of *Citrobacter freundii*, a red-leg syndrome associated pathogen in raniculture, by indigenous *Lactococcus lactis* CRL 1584. Vet Microbiol 151:336–344. 10.1016/j.vetmic.2011.03.025 21531092 10.1016/j.vetmic.2011.03.025

[CR16] Pessier AP (2017) Hopping over red leg: the metamorphosis of amphibian pathology. Vet Pathol 54:355–357. 10.1177/0300985817699861 28438113 10.1177/0300985817699861

[CR17] Posada D, Crandall KA (1998) MODELTEST: testing the model of DNA substitution. Bioinformatics 14:817–818. 10.1093/bioinformatics/14.9.817 9918953 10.1093/bioinformatics/14.9.817

[CR18] Ribeiro TG, Goncalves BR, da Silva MS, Novais Â, Machado E, Carrico JA, Peixe L (2017) *Citrobacter portucalensis* sp. nov., isolated from an aquatic sample. Int J Syst Evol Microbiol 67:3513–3517. 10.1099/ijsem.0.002154 28857032 10.1099/ijsem.0.002154

[CR19] Ronquist F, Huelsenbeck JP (2003) MrBayes 3: Bayesian phylogenetic inference under mixed models. Bioinformatics 19:1572–1574. 10.1093/bioinformatics/btg180 12912839 10.1093/bioinformatics/btg180

[CR20] Sai S, Mani R, Ganesan M (2023) Isolation and identification of *Citrobacter* species. In: Thomas J, Amaresan N (eds) Aquaculture Microbiology. Springer US, New York, NY, pp 29–35. 10.1007/978-1-0716-3032-7_4

[CR21] Sambrook J, Fritsch EF, Maniatis T (2001) Molecular cloning: A laboratory manual. Cold Spring Harbor, Cold Spring Harbor Laboratory, Cold Spring Harbor, New York

[CR22] Sellera FP, Fernandes MR, Fuga B, Fontana H, Vásquez-Ponce F, Goldberg DW, Monte DF, Rodrigues L, Cardenas-Arias AR, Lopes R, Cardoso B, Costa DGC, Esposito F, Lincopan N (2022) Phylogeographical landscape of *Citrobacter portucalensis* carrying clinically relevant resistomes. Microbiol Spectr 10:e01506-e1521. 10.1128/spectrum.01506-21 35357225 10.1128/spectrum.01506-21 PMC9045157

[CR23] SEMARNAT. 2010. Norma Oficial Mexicana NOM-059-SEMARNAT-2010, Protección ambiental - Especies nativas de México de Flora y Fauna Silvestres-Categorías de Riesgo y especificaciones para su inclusión, exclusión o cambio–Lista de Especies en Riesgo. Diario Oficial de la Federación 2a Sección, 30 de diciembre del 2010. https://www.dof.gob.mx/normasOficiales/4254/semarnat/semarnat.htm. Accessed 30 October 2024

[CR24] Smith JE, Rojas LA (2023) What it takes to save the axolotl. Int. NYT*,* (2023, December 8). https://www.nytimes.com/2023/12/05/science/mexico-axolotl-biology.html. Accessed 30 October 2024

[CR25] Soto-Cortés E, Marroquín-Rodríguez M, Basanta MD, Maldonado-López Y, Parra-Olea G, Rebollar EA (2024) Host species and snvironment shape the skin microbiota of Mexican Axolotls. Microb Ecol 87:98. 10.1007/s00248-024-02411-1 39046491 10.1007/s00248-024-02411-1 PMC11269437

[CR26] Takami Y, Une Y (2017) A retrospective study of diseases in Ambystoma mexicanum: a report of 97 cases. J Vet Med Sci 79:1068–1071. 10.1292/jvms.17-0066 28529268 10.1292/jvms.17-0066 PMC5487785

[CR27] Tekedar HC, Kumru S, Blom J, Perkins AD, Griffin MJ, Abdelhamed H, Karsi A, Lawrence ML (2019) Comparative genomics of *Aeromonas veronii*: identification of a pathotype impacting aquaculture globally. PLoS ONE 14:e0221018. 10.1371/journal.pone.0221018 31465454 10.1371/journal.pone.0221018 PMC6715197

[CR28] Thompson JD, Higgins DG, Gibson TJ (1994) CLUSTAL W: Improving the sensitivity of progressive multiple sequence alignment through sequence weighting, position-specific gap penalties and weight matrix choice. Nucleic Acids Res 22:4673–4680. 10.1093/nar/22.22.4673 7984417 10.1093/nar/22.22.4673 PMC308517

[CR29] Wang B, Hu J, Feng J, Zhang Y, Sun Y, Jiang B, Li W, Liu C, Huang Y, Su Y (2022) Acute septicemia and immune response of spotted sea bass (*Lateolabrax maculatus*) to *Aeromonas veronii* infection. Fish Shellfish Immunol 124:47–55. 10.1016/j.fsi.2022.03.030 35367379 10.1016/j.fsi.2022.03.030

[CR30] Yang P, Zheng Y, Zou X, Sun Y, Liu Y (2024) Comparative transcriptomic analysis of gene expression profiles in the liver and spleen of American bullfrog (*Lithobates catesbeianus*) in response to *Citrobacter freundii* infection. J World Aquac Soc 55:353–372. 10.1111/jwas.12999

[CR31] Zepeda-Velazquez AP, Gómez-De-Anda FR, Aguilar-Mendoza LF, Castrejón-Jiménez NS, Hernández-González JC, Varela-Guerrero JA, de la Rosa-Arana JL, Vega-Sánchez V, Reyes-Rodríguez NE (2023) Bullfrogs (*Lithobates catesbeianus*) as a potential source of foodborne disease. J Food Protect 86:100067. 10.1016/j.jfp.2023.100067 10.1016/j.jfp.2023.100067 36948016

